# Real-time pathologist-assisted field postmortem examinations of beef cattle

**DOI:** 10.1177/10406387241269043

**Published:** 2024-08-17

**Authors:** Jennifer L. Davies, Lindsay Rogers, Dayna Goldsmith, Grace P. S. Kwong, Carolyn Legge, Erin Zachar

**Affiliations:** Faculty of Veterinary Medicine, University of Calgary, Calgary, Alberta, Canada; Faculty of Veterinary Medicine, University of Calgary, Calgary, Alberta, Canada; Faculty of Veterinary Medicine, University of Calgary, Calgary, Alberta, Canada; Faculty of Veterinary Medicine, University of Calgary, Calgary, Alberta, Canada; Department of Community Health Sciences, Cumming School of Medicine, University of Calgary, Calgary, Alberta, Canada; Faculty of Veterinary Medicine, University of Calgary, Calgary, Alberta, Canada; Faculty of Veterinary Medicine, University of Calgary, Calgary, Alberta, Canada

**Keywords:** cattle, laboratories, pathologists, postmortem examination, telepathology, veterinarians

## Abstract

Postmortem examination of deceased production animals with appropriate ancillary testing is fundamental to determining causes of morbidity and mortality. Reaching a definitive diagnosis is crucial to evidence-based herd management and treatment decisions that safeguard animal health and welfare, food safety, and human health. However, for a range of reasons, carcasses sometimes cannot be examined in a veterinary diagnostic laboratory. As a result, postmortem examinations of farmed animals, including cattle, are often performed on-farm by the referring veterinarian (rVet) with tissue samples submitted to a veterinary diagnostic laboratory for ancillary testing. For various reasons, field postmortems can be associated with lower diagnostic rates. We investigated real-time pathologist-assisted field postmortem examination (rtPAP) assistance to beef cattle rVets to gauge any improvement in attaining a final diagnosis. We found that rtPAPs improved the success of reaching a final diagnosis compared to unassisted field postmortem examinations. Both the participating bovine rVets and the pathologists saw benefits to the rtPAPs, with bovine rVets indicating that they would utilize this service in the future if available. Our proof-of-concept study demonstrated the positive role of rtPAPs in diagnosing beef cattle disease and speaks to the need for telepathology services supporting food animal rVets and producers.

Postmortem examinations (“postmortems”) of deceased production animals with appropriate ancillary testing are essential tools to determine causes of morbidity and mortality. An accurate diagnosis informs management practices, optimizes treatment to mitigate further losses in a herd, provides early warning of outbreaks, and delivers information to surveillance groups.^16^ Postmortems are typically performed by veterinary pathologists (“pathologists”) when whole bodies are submitted to a veterinary diagnostic laboratory. However, there are many factors that influence veterinarians’ and producers’ decisions to submit samples to diagnostic laboratories, such as distance to the lab, difficulty in transporting large carcasses, cost, negative previous experiences with the lab, such as slow turnaround times or poor communication, and previous inconclusive results.^6,9,11,12,14^ Given these barriers, and others, the estimate is that <1% of cattle that die before slaughter undergo postmortems, which is a gap in surveillance.^1^ To eliminate some barriers to submission, most notably the distance to the lab, postmortems of cattle can be performed on-farm by the referring veterinarian (rVet) with submission of formalin-fixed and fresh tissue samples to the laboratory for additional testing and diagnosis.^1^ These field postmortem submissions are associated with a lower diagnostic rate, and the failure to reach a diagnosis can leave the producer, rVet, and pathologist dissatisfied, which undermines the value of the postmortem examination in disease investigation.^4,15^

The Diagnostics Services Unit (DSU) in the Faculty of Veterinary Medicine at the University of Calgary (UCVM; Calgary, Alberta, Canada) receives both whole bodies for postmortems and tissue samples from field postmortems for analysis. Anecdotally, pathologists at the DSU observed that a definitive diagnosis was reached more often when a whole body was received for postmortem examination versus tissues from a field postmortem. This discrepancy is supported by the literature wherein the submission of whole carcasses, compared to non-carcass tissue submissions and partial postmortems, is a significant positive factor in determining a diagnosis.^4,15^

Telepathology is not a new phenomenon, but since ~2015, significant advances in information technology and telecommunications coupled with the pandemic have led to unprecedented sophistication, accessibility, and use in human and veterinary medicine.^3^ With current video and cellular data technology, pathologists could assist rVets in the field with postmortems through telepathology. In veterinary medicine, telepathology has largely been restricted to digital microscopy for cytology, hematology, and histopathology.^8,13^ For gross pathology in cattle, the development of a standardized postmortem examination procedure with digital photographs often performed by a technical staff member, called *remote digital necropsy*, has been developed to allow a rVet or pathologist to assess the body after the postmortem is completed.^1,5^ Although still images for gross anatomic telepathology or “digital necropsies” certainly have utility, they are associated with significant limitations including the static nature of the images and lag time between the postmortem and the examination of the digital photographs. Not all organ systems may be examined, photographs may be biased by the prosector’s interpretation, and appropriate samples may not have been harvested for ancillary testing.^13^ The use of video for *real-time pathologist-assisted field postmortems* (rtPAPs) would allow the pathologist to see the body in situ and consult on tissue sampling to improve the likelihood of coming to a final diagnosis from a field postmortem. A 2024 scoping review of telepathology showed a knowledge and research gap for rtPAPs in veterinary medicine, although some work has assessed the use of augmented reality for meat inspection.^2,13^

Our objectives were to 1) determine if rtPAPs improve the success of coming to a final diagnosis compared to unassisted field postmortems, and 2) determine if rtPAPs are a useful and viable service option to be offered to food animal veterinary practitioners.

## Materials and methods

### Animal care protocols and ethics

Our study was reviewed and approved by the Conjoint Faculties Research Ethics Board (CFREB), University of Calgary (certification REB18-1531) and the Animal Care Committee, University of Calgary (certification AC22-0031).

### Participants

We contacted 10 veterinary clinics with high caseloads of beef cattle via email and invited them to enroll in our study. These clinics regularly submitted beef cattle samples to the DSU. Five of the clinics accepted and enrolled in the study. Four ACVP board-certified veterinary pathologists working in the DSU agreed to participate in the study.

### Sample size

We calculated sample size as 40 field postmortems per group for the paired (before–after) design after adjustment for clustering with an assumed interclass correlation (ICC) of 0.15, alpha = 0.05, power = 0.80. A total of 80 field postmortems were to be performed: 40 without video assistance and 40 with video assistance. Each veterinary clinic was allotted field postmortems proportionate to the number of beef cattle rVets at their clinic and equal numbers of unassisted and assisted field postmortems. We covered the cost of a routine field postmortem submission to the DSU plus 2 ancillary tests (as chosen by the pathologist) to ensure that cost did not influence case selection or diagnostic outcome. No additional costs were charged to the rVet for the rtPAP.

### Case selection

The beef cattle rVet chose the cases for enrollment with an effort to reflect typical cases requiring field postmortem examination. Eligibility requirements were beef cattle ≥48-h-old given that bovine abortions, stillbirths, and early neonatal deaths have inherently low diagnostic rates.^4,15^

### Unassisted postmortem examination procedure

Our study consisted of 2 parts over 13 mo from April 2022 to May 2023. In the first part of the study (Apr 2022–Jan 2023), field postmortems performed by each enrolled rVet were not assisted by a pathologist. rVets submitted field postmortem samples to the DSU as a routine submission, following their standard approach. We provided the enrolled rVets with field postmortem kits containing sample containers, shipping labels, and DSU bovine submission forms. The submission was assigned to the DSU duty pathologist on the day the sample arrived. rVets were not informed about future video assistance for their field postmortems to avoid influencing their postmortem and tissue submission decisions.

### Assisted postmortem examination procedure

In the second part of our study (Feb–May 2023), field postmortems were pathologist-assisted in real-time by one of the participating DSU pathologists. We provided the rVets with the same field postmortem kits as in the first half of the study. The rtPAPs were scheduled during regular DSU business hours for a time that worked for the pathologist, rVet, and producer. The rtPAPs used the Google Meet video conferencing system (https://workspace.google.com/products/meet/) either with the rVet’s cell phone mounted on a tripod with full view of the animal or an assistant holding the phone for the field postmortem.

The rVet and pathologist began their call with a discussion of animal signalment and history. During the rtPAP, the pathologist and rVet worked through the field postmortem together in real time. The pathologist was able to request examination of specific body systems as well as collection of specific formalin-fixed and fresh tissue samples. Ancillary tests were also discussed. The rVets and pathologists followed a systematic postmortem examination procedure, with modifications specific to each case depending on case signalment, history, and differential diagnoses.^7^ Field postmortem samples (formalin-fixed and chilled) arrived (dropped off by the clinic or a courier) to the DSU within 1 business day of the postmortem, and the pathologist who assisted the field postmortem also performed the histopathology and directed the ancillary testing for that case (Suppl. Doc. 1).

### Data collection

Samples were received and processed in accordance with DSU SOPs (information available upon request). We stored case data in the DSU’s secure Laboratory Information Management System (LIMS; Vetstar Animal Disease Diagnostic System [VADDS], Advanced Technology) as required for all cases submitted to the DSU. We anonymized the case data and recorded the case type (unassisted or rtPAP), rVet and clinic, case pathologist, animal signalment, history, pathology diagnoses, final diagnoses, diagnostic testing cost, and whether a final diagnosis was reached (outcome). Our outcome was categorized into reaching a final diagnosis or a no or partial diagnosis. Establishing a definitive diagnosis is typically the ultimate goal in examination of a specimen and often requires ancillary testing to determine an etiology. We defined *definitive diagnosis* as the identification of a specific disease or cause of the lesions.^10^ Cases in which a definitive diagnosis was reached for all morphologic diagnoses were classified as reaching a final diagnosis. Cases with more than one morphologic diagnosis, in which not all definitive diagnoses were determined, were recorded as having reached a no or partial diagnosis. For example, an animal with diarrhea caused by necrotizing and hemorrhagic typhlocolitis caused by coccidiosis also had histologic lesions suggestive of septicemia. However, no fresh tissue was available for culture and, therefore, the etiologic agent was not established, and this case was recorded as reaching a partial diagnosis. Each rVet, clinic, and pathologist was assigned a random identifying number to ensure anonymity of their cases and identities.

We surveyed participating rVets and pathologists following the study and anonymized the responses to provide qualitative information about the viability of rtPAP as a service (Suppl. Docs. 2, 3). The survey was all free-text responses.

### Data analysis

We completed the statistical analysis using R (https://www.r-project.org). We presented summary statistics using count with percentage for categorical data including case type, definitive diagnostic result, and their distributions by clinics and pathologists.

We used a logistic regression model to investigate the likelihood of reaching a definitive diagnosis with and without rtPAP. In addition, we examined whether the individual rVets or case pathologists had an effect on the outcome using analysis of deviance.

## Results

Fifteen rVets from 5 clinics enrolled in our study, and they completed 58 field postmortems: 32 unassisted field postmortems and 26 rtPAPs (Table 1). One veterinary clinic did not perform any rtPAPs, and no reason was given for withdrawal from the study after the first half. To increase our sample size, their unused rtPAP allotment was given to another participating clinic in the last 2 wk of the study. One clinic only performed one rtPAP because the lead rVet for the clinic was away for most of the second half of the study.

**Table 1.** Number of unassisted field postmortems and real-time pathologist-assisted field postmortems (rtPAPs) by clinic and referring veterinarian (rVet).

Veterinary clinic	rVet ID	No. unassisted field postmortems	No. rtPAPs	Total no. field postmortems by clinic
1	2	6	12	29
	8	2	0	
	9	2	0	
	10	1	3	
	11	1	0	
	15	0	2	
2	4	3	0	8
	6	1	0	
	14	0	4	
3	1	6	1	10
	2	1	0	
	3	2	0	
4	7	1	0	4
	12	2	0	
	13	1	0	
5	5	3	4	7
Total	NA	32	26	58

ID = identifier; NA = not applicable.

Of the unassisted field postmortems, 21 of 32 (66%) reached a final diagnosis. Of the assisted field postmortems, 25 of 26 (96%) reached a final diagnosis. The odds of reaching a final diagnosis with rtPAP were 13.1 (2.27–249; *p* = 0.018) times that of reaching a final diagnosis without rtPAP (Table 2). Even though one rVet performed 46% of the rtPAPs (Table 1), reaching a final diagnosis was not significantly impacted by which rVet (*p* = 0.32) or case pathologist (*p* = 0.17) was involved in the submission.

**Table 2.** Cases with and without a final diagnosis for unassisted field postmortems and real-time pathologist-assisted field postmortems (rtPAPs).

	No or partial diagnosis	Final diagnosis reached	Total	Odds ratio (95% CI)	*p*
Unassisted field postmortem	11 (34%)	21 (66%)	32	13 (2–249)	0.018
rtPAP	1 (4%)	25 (96%)	26		
Total	12	46	58		

Three rVets from 3 different clinics returned completed surveys; the results were generally positive. All 3 respondents found assistance during postmortems beneficial to their practice and would utilize the service were it offered by the DSU, especially for more challenging cases. They also valued discussing cases in real-time with the pathologist. All 3 rVets indicated that the assisted postmortems improved their field postmortem skills by improving tissue sampling procedures, identifying lesions that may have gone unnoticed, or becoming more systematic in the procedure. Challenges identified by practitioners were scheduling a pathologist for a call as rVets often work outside of typical university business hours and technology issues such as difficulty arranging the camera and connectivity while on-farm.

All 4 pathologists who provided assisted postmortems returned completed surveys and indicated that assisting during field postmortems was beneficial to their diagnostic work-up of the case. Two of the pathologists specifically indicated that they felt that rtPAPs improved their efficiency in working up these cases and allowed them to interpret the histopathology more quickly. The primary advantages identified by pathologists were increased likelihood of receiving all of the samples needed to arrive at a final diagnosis, ability to gather important clinical information while on the phone with the rVet, developing a relationship between the rVet and the pathologist, and increased exposure to what is common in practice to improve teaching of veterinary students. One pathologist noted that rtPAPs could improve the safety of the rVet working alone in the field. The major challenge identified by the pathologists was poor image quality, especially in areas of poor connectivity or camera movement that would pixilate the video. One pathologist noted that having an owner present made it more difficult to ask the rVet questions that may appear as questioning the rVet’s knowledge in front of the owner. Overall, all participating pathologists were willing to provide the service to rVets in the future.

## Discussion

With ever-improving telecommunications, the use of telepathology in veterinary medicine is increasing. A 2024 scoping review indicated that the value of video for rtPAPs for diagnostic cases had not been explored in the literature.^13^ Our results provide valuable insight into the viability of offering rtPAPs as a service to beef cattle producers and rVets. In our study, rtPAPs were associated with a higher diagnostic rate than the unassisted field postmortems. Furthermore, almost 10% of unassisted cases did not reach a morphologic diagnosis let alone a final diagnosis; this challenge was not encountered in the assisted cases. Even though this difference was not statistically significant between the groups, it is an interesting observation as these inconclusive case results could contribute to client dissatisfaction. Non-carcass tissue submissions are known to be associated with lower diagnostic rates.^4,15^ The literature also indicates that previous inconclusive results decrease the likelihood of future sample submission by producers and rVets and that positive case outcomes are a high priority for cattle veterinarians.^11,12,14^

Our study demonstrated the desire of rVets to use this service and the willingness of pathologists to deliver the service, highlighting the value of telepathology. In addition to an improved diagnostic rate, continuing education and relationship-building were highlighted as positive outcomes in our study. In a previous study, practitioners identified a lack of confidence in sample-taking, and this uncertainty made them less likely to submit samples to the laboratory.^12^ Therefore, rtPAPs could be an important tool in outreach and continuing education to improve practitioner knowledge around tissue sampling and submission. The service could also be extremely valuable in training veterinary students, in supporting newly graduated practitioners in the field, or in guiding veterinary technicians. Several studies have investigated the importance of a positive relationship between the rVet and laboratory in increasing likelihood of sample submission.^11,12^ rtPAPs improve personal communication with virtual face-to-face interaction between the rVets and pathologists, increasing trust and confidence in the diagnostic process. Although our study demonstrated increased diagnostic rates and a willingness for rVets and pathologists to engage in rtPAPs, we did not examine the economic viability and long-term sustainability of this service. Further analysis to assess the long-term economic viability of rtPAP services and creation of a business model, including fee structure and ongoing use by rVets, are warranted and may vary between laboratories and geographic regions.

rtPAPs are not without their challenges. The service’s availability is highly dependent on telecommunications services in the area, and lack of telecommunication services may limit use in remote areas. Even in areas with telecommunication support, issues such as adverse weather conditions, poor video, poor audio, and equipment setup were cited as challenges by both rVets and pathologists in our study. Some of these challenges will hopefully be negated as technology advances. An additional issue identified by practitioners both in our study and in the literature is the lack of access to diagnostic expertise outside of regular laboratory business hours.^12^ rtPAPs alone cannot eliminate this barrier.

As a proof-of-concept study, our low case numbers resulted in statistical analysis with wide CIs. Low case numbers were partly due to seasonal variation in the production cycle of beef cattle in Alberta and to slow uptake of the service early in the study. We also approached clinics already known to the DSU for participation and one rVet performed almost half of the rtPAPs. These factors had the potential to introduce bias into the study, although the outcome (reaching a final diagnosis) was not affected by the rVet or the pathologist. Future studies should investigate whether similar improvements in diagnostic rates are achieved across veterinary or pathology teams with various levels of experience. Further exploration of this service with other production animal species and the use of rtPAPs for continuing education and laboratory outreach are warranted.

The higher rate of diagnosis associated with rtPAPs that we found could translate into better use by producers of veterinary resources; improved satisfaction for producers, rVets, and diagnosticians; and increased awareness of the value of postmortems for diagnosis of animal disease. More broadly, this service would better support animal disease surveillance leading to improved animal health and welfare, food safety, market access, consumer confidence, and public health. And finally, this technology could extrapolate beyond cattle testing to support all on-farm food animal species testing.

## Supplemental Material

sj-pdf-1-vdi-10.1177_10406387241269043 – Supplemental material for Real-time pathologist-assisted field postmortem examinations of beef cattle Supplemental material, sj-pdf-1-vdi-10.1177_10406387241269043 for Real-time pathologist-assisted field postmortem examinations of beef cattle by Jennifer L. Davies, Lindsay Rogers, Dayna Goldsmith, Grace P. S. Kwong, Carolyn Legge and Erin Zachar in Journal of Veterinary Diagnostic Investigation
