# Supplementary material for: Real-time pathologist-assisted field postmortem examinations of beef cattle
Source: J Vet Diagn Invest. 2024 Aug 17;36(6):799–803. doi: 10.1177/10406387241269043 (PMC11529060; doi:10.1177/10406387241269043)
Supplement: sj-pdf-1-vdi-10.1177_10406387241269043 – Supplemental material for Real-time pathologist-assisted field postmortem examinations of beef cattle [file sj-pdf-1-vdi-10.1177_10406387241269043.pdf]

JVDI: Supplemental material

Davies JL, et al. Real-time pathologist-assisted field postmortem examinations of beef cattle

Supplemental Document 1. Procedure for real-time pathologist-assisted field postmortem examinations (RT-PAFPs)

1. Participating veterinarian ("veterinarian") identifies an eligible case for inclusion in the study. Eligible cases are the same as for the unassisted field postmortem examinations: beef cattle 48 hours and older. Abortions, non-viable neonates, and neonatal deaths under 48 hours of age are not eligible.
2. Veterinarian contacts the Diagnostic Services Unit (DSU) reception desk via phone call (**XXX-XXX-XXXX**) as soon as they identify the case and within the DSU's regular hours of operation. *They confirm that they have adequate cell reception at the RT-PAFP location for a video call* and a field necropsy kit provided to them by the research study. **Google Meet is the most reliable app for poor cell reception.**
    a. RT-PAFPs will only occur within the DSU's regular hours of operation: M-F, 8:30am-4:30pm (closed weekends and statutory holidays)
3. DSU reception will arrange a meeting time for the on-call veterinary pathologists ("pathologist") and veterinarian. This will be a video call via Google Meet.
4. Once connected, the veterinarian and pathologist will have a brief discussion about signalment, history, and differential diagnoses of concern before starting the RT-PAFP.
5. The veterinarian will then mount their phone using the tripod provided, with the animal carcass in full view of the camera. It is ideal if the animal is close to the object holding the tripod with the phone pointed down at the animal as much as possible. Alternatively, the veterinarian can have an assistant hold the phone.
    a. The veterinarian will have time before starting the RT-PAFP to don personal protective equipment (PPE) and organize their postmortem examination equipment as necessary.
6. The pathologist will direct the veterinarian through the RT-PAFP.
    a. The veterinarian may be asked to describe tissues to the pathologist during the call as it will likely be difficult to manipulate the video during the call to zoom in on particular tissues.
    b. Please note: it was decided to not create an exact protocol for the RT-PAFPs as the focus of the necropsy will change depending on case signalment, history, and differential diagnoses. It is expected that all body systems will be covered.
7. Tissues selected for submission to the DSU for further processing and diagnostics will be packaged for shipment using the field postmortem kit provided to the veterinarian by the research team. The submission will include a completed DSU Bovine Submission Form.
    a. Tissues selected for submission to the DSU may vary from case to case. They will be selected with input from both the pathologist and veterinarian.

8. Before ending the video call, the veterinarian will be given a chance to further discuss the case with the pathologist.
9. Tissues will be submitted to the DSU for processing and diagnostics and findings will be reported back to the veterinarian following usual DSU procedures. The veterinarian is responsible for reporting back to the animal owner.
    a. The veterinarian is welcome to contact the pathologist at any point in the diagnostic process to discuss the case. This is routine procedure at the DSU.

Appendix A. Procedure for scheduled RT-PAFPs with inadequate cell reception

In the event of inadequate cell reception for a video call at the RT-PAFP location, the following options are available.

Option 1: Troubleshooting – try a different method of connecting or connect to farm Wi-Fi if available

- Google Meet and Zoom appear to be more reliable in areas of poor reception than FaceTime or MS Teams.
- Recognizing that the time of the pathologists and veterinarians is valuable, only 5-10 minutes should be spent on this option.

Option 2: Regular phone call

- The pathologist calls the veterinarian on their cell phone. The veterinarian describes findings over phone while the pathologist helps direct sampling. If possible the veterinarian should take photos for submission with postmortem tissues.
- Veterinarian receives full reimbursement for time and the RT-PAFP counts towards allotted number.

Option 3: Abandon RT-PAFP

- Veterinarian proceeds with their regular field postmortem without additional assistance.
- Tissues are submitted at discretion of veterinarian but may not be covered by study. If funds are available, the study will cover the costs as an unassisted field postmortem, otherwise the submission will be billed back to clinic.
- No reimbursement for the field postmortem and the RT-PAFP does not count towards allotted number

Appendix B. Instructions for using Google Meet

Google Meet is a free app for video calling available on both Android and iOS devices. It claims to be the most reliable for areas with poor connectivity.

1. Download Google Meet from your phone’s app store.
2. Sign in with a personal email account, or use research study account:
    a. Email: XXXXXXX@gmail.com
    b. Password: xxxxxxxx
3. Join meeting with link or code provided by the DSU.

4. (From a web browser: https://meet.google.com → meeting code (or click meeting link provided) → enter name and wait to be admitted to the call)
    a. Most phones will direct you to the app when clicking this link and will not allow you to connect via web browser.

Supplemental Document 2. Questionnaire for participating beef cattle veterinarians

1. Did you find assistance during field postmortem examinations beneficial to your practice?
2. Please provide your thoughts on the positives and negatives of the real-time pathologist-assisted field postmortem examinations (RT-PAFPs).
3. Please comment on the technology used (video call via cell phone) for the RT-PAFPs and any suggested improvements to the RT-PAFPs.
4. Would you use RT-PAFPs in the future if they were a service offered by the Diagnostic Services Unit (DSU)?
5. Please provide any other comments on the RT-PAFPs.

Supplemental Document 3. Questionnaire for participating pathologists

1. Did you find assisting during field postmortem examinations beneficial to your diagnostic work-up of the submitted tissues?
2. Please provide your thoughts on the positives and negatives of the real-time pathologist-assisted field postmortem examinations (RT-PAFPs).
3. Please comment on the technology used (video call via cell phone to a computer monitor) for the RT-PAFPs and any suggested improvements to the RT-PAFPs.
4. Would you be willing to provide RT-PAFPs to practitioners in the future if they were a service offered by the Diagnostic Services Unit (DSU)?
5. Please provide any other comments on the RT-PAFPs.
